# A new approach to analysis of relationships between ^137^Cs activity concentrations in forest soil horizons

**DOI:** 10.1007/s10967-013-2828-9

**Published:** 2013-11-14

**Authors:** Zbigniew Ziembik

**Affiliations:** Independent Chair of Biotechnology and Molecular Biology, Opole University, ul. kard. B. Kominka 6, 45-032 Opole, Poland

**Keywords:** ^137^Cs, Forest soil, Distribution, Compositional data

## Abstract

The measurements results of ^137^Cs activity concentrations in forest soil profiles are discussed. In studies some simplifications were considered. First of them concerns disregarding of soil subtype in data analysis. However initially this parameter was considered in data analysis, it was finally ignored. The second assumption drops information about specific soil horizon. Description of ^137^Cs accumulation is based on relationships between its relative activity concentrations in soil layers. The model formulation was based on the results of exploratory data analysis of the relative ^137^Cs activity concentrations. In studies the methods designed for compositional data analysis were used. The results of analysis showed that the relationships between the relative activity concentrations of ^137^Cs in soil layers, due to their nature, might be divided into two groups. The first of them concerns layers located close to soil surface. The relative activity concentrations of ^137^Cs in these layers are proportional to each other, and distribution mechanism of cesium within them has the characteristics of the process leading to thermodynamic equilibrium. The second group is related to layers that are located deeper. The calculation results suggest lack of thermodynamic equilibrium between these layers and layers situated above. Utilization of a linear model for description of changes in relative activity concentrations of ^137^Cs in deeper layers supposes that these changes occur much slower than in layers lying above.

## Introduction

A large amount of different artificial radionuclides appeared in environment as a result of nuclear weapon tests, conducted especially frequently in the middle of the 20th century. Environment was contaminated also as a result of a number of incidental, uncontrolled releases [1–3], like for example, accidents in Chernobyl (1986) and Fukushima Dai-Ichi (2011) nuclear power plants [4–8]. As a result, within a period of days and weeks, radioactive dust or contaminated water was dispersed in long distances, reaching all or almost all continents [9–12].

Significant quantities of radioactive matter were detected also in regions where certain types of industry were located. Particularly nuclear facilities used for nuclear fuel or weapon production can be a source of radioactive contamination, not only in their vicinity but also in distant areas [13]. Improper maintenance or execution of procedures which disregards security measures may also lead to uncontrolled penetration of dangerous radioactive materials into environment [14].

Among artificial radionuclides which can be found in environment, the ^137^Cs isotope requires special attention. It is produced during nuclear bomb explosion and is a product of processes occurring in nuclear reactor.

Relatively long half-life time and chemical properties of ^137^Cs, which promote its penetration to food chains, calls special interest in monitoring of this isotope presence in environment. In land environment radionuclides, sooner or later, find their way to soil.

Because of the structure, chemical composition and physical properties of soil, migration of a substance in this environment is a complex process, and its description can be only approximate [15, 16]. For example, the ^137^Cs activities of concentration in forest soil horizons can be partly predicted on the base of soil physicochemical properties [17–21].

The main component contributing in a substance transport in soil is water. In simplified approach soil can be regarded as a porous medium unsaturated or partially saturated with water. There are three basic transport mechanisms in such medium: molecular diffusion, hydrodynamic dispersion and advection [22–24].

Usually, the assumption in description of substance migration is transport of dissolved or dispersed in liquid phase material from the surface to deeper soil regions. The dispersion-advection models of transport in soil usually requires homogeneity of the soil structure [25, 26]. Dispersion coefficient and advection velocity are assumed to be constant in all the soil profile. The approximation of uniform transport mechanisms in soil profile can be omitted introducing parameters that are related to changes in physicochemical properties of soil during penetration of solution into deeper regions. In some dispersion-advection models the specificity of interactions between components of solid and liquid phases are considered. For example, the distribution coefficient describing the ratio of a substance concentration in soil solution and in soil matrix was introduced to model [27]. Some transport models include parameters related to surface vegetation and water budget in soil [28].

The compartment models are also used for description of substance migration in soil. It can be assumed that soil is composed of set of layers and a matter transport occurs between them. Each layer is regarded as separate compartment, with its own specific properties. The compartments can be soil layers or genetic soil horizons, dependently on the initial presumptions of the model [25, 29].

It is expected that the processes determining distribution of ^137^Cs in soil are related to chemical and physical properties of solid, liquid and gaseous soil components. Biological activities and atmospheric phenomenons also affect this distribution. A variety of factors influencing transport and retention mechanisms and their considerable randomness suppose possibility of partial neutralization of their impacts on ^137^Cs distribution. As a result it should be possible to determine an effect of resultant factors impact, even if their nature would not be recognized. Reducing the number of parameters in description of cesium accumulation could provide information about the basic mechanism, that are not determined strictly by the local soil properties.

One of the most discernible properties of forest soils is their structure consisting of distinguishable layers. In soil profile they appear in a sequence and in model assumptions they can be regarded as a homogeneous, effective medium in which transport and retention of ^137^Cs occurs. Though properties of these layers depend on soil type, their sequence in soil profile could be regarded as the most important discriminating factor. Despite that the layers are recognized as specific soil horizons or subhorizons, the adjacent layers also share some common features. For example, the upper layers are rich in organic matter while in the lower ones the mineral matter prevails.

An effort could be undertaken to describe distribution of ^137^Cs in soil and to analyze relationships between this radioisotope concentration in layers. As a result an information about transport mechanisms which are not strictly bound with peculiar properties of soil profile components could be perceived.

In this paper description of ^137^Cs accumulation in soil is based on the relationships between its activity concentrations in soil layers. The model formulation is based on the results of exploratory data analysis. In the measurement results interpretation the methods designed for compositional data analysis were used. The formulated approach to construction of the model of ^137^Cs distribution in soil is new, and it was not described earlier.

## Sampling methods and computations

In computations the data obtained in measurements of ^137^Cs activity concentrations in samples of genetic horizons of forest soil, which were collected in south-western Poland and in the Polish–Czech border region in the area of so called Opole Anomaly, were used. The soil horizon is the specific layer in soil, parallel to the land surface and possessing physical characteristics different from the layers above and beneath. The soil profile samples were collected in autumn, when vegetation season was finished.

In the collected samples of soil profiles the O, A, E, B and C master profiles were identified. Some characteristic features of soil horizons could be mentioned. The O horizons comprise surface layers dominated by organic material continuously or periodically saturated with water. Organic material is observed in different decay stages, increasing from Ol, through Of, to Oh subhorizon. In forest soil the mineral A horizons is formed below an O horizon. They exhibit obliteration of all or most of the original rock structure and show an accumulation of humified organic matter mixed with the mineral fraction. They are not dominated by properties characteristic of E or B horizons. In mineral E horizons, similarly like in A, the original rock structure is obliterated. The main feature is the loss of silicate clay, iron, aluminum, preserving concentration of sand and silt particles. The B horizons are formed below an A, E, or O horizon. They are also dominated by the obliteration of the original rock structure. Among others, in B horizons illuvial concentration of some components (silicate clay, iron, aluminum, carbonates, gypsum), evidence of the removal or addition of carbonates and residual concentration of oxides can be observed. The C horizons are usually mineral, and they are little affected by pedogenic processes. They do not posses the properties of O, A, E, or B horizons [30].

The samples were taken in the vicinity of at least 20 years old trees, not less than 100 m from roads. On the ground around the trees grew at most few forest bed plants, it was covered mainly by fallen tree leaves. In investigations the soil profiles composed of at least 5 soil horizons and subhorizons were considered. The results from 39 soil profiles were used in analysis.

In soil profile samples different horizons and subhorizons were recognized. However the data for Ol, Of, Oh, A, Bbr, Ees and C soil horizons have been already presented and used in investigation of relationships between the horizons physicochemical properties and the relative ^137^Cs concentrations [17, 31], some previously unpublished measurement results obtained for OhA, AE and Abbr horizons were included in current studies.

The samples of soil horizons were taken from the places, where the following soil types were identified: podzols (*PZ*), brown soils (*CM*), mixed gley soils (*GLm*), podzol soils proper (*PLp*), pseudogley soils (*Gls*), soils lessives typical (*LV*), rankers brown (*LC*), rendzinas brown (*RB*). The width of a soil horizons usually varied from about 0.5 cm to 2.5–3 cm. The thickest was the C horizon, from which the column shaped sample with height of approx. 5–6 cm was taken. The soil profiles were usually composed of four to six horizons and the identified horizon types were often different. The maximum depth of soil penetration reached about 30 cm. It was found that all or nearly all ^137^Cs activity was spread within this depth.

The measurement of ^137^Cs activity in samples of soil horizons was carried out by means of a gamma-spectrometer with a germanium detector HPGe (Canberra) of high resolution: 1.29 keV (FWHM) at 662 and 1.70 keV (FWHM) at 1,332 keV. Relative efficiency: 21.7 %. Energy and efficiency calibration of the gamma spectrometer was performed with the standard solutions type MBSS 2 (Czech Metrological Institute, Prague), which covers an energy range from 59.54 to 1,836.06 keV. The geometry of the calibration source was a Marinelli container (447.7 ± 4,5 cm^3^), with density 0.985 ± 0.01 g/cm^3^, containing ^241^Am, ^109^Cd, ^139^Ce, ^57^Co, ^60^Co, ^137^Cs, ^113^Sn, ^85^Sr, ^88^Y and ^203^Hg. The geometry of sample container was a similar Marinelli of 450 cm^3^. Time of measurement was 24 h for all of soil horizon samples. Measuring process and analysis of spectra were computer controlled with use of the software GENIE 2000. The results were corrected to the same date of measurement.

For statistical computations the R language [32] was utilized. R is a free software environment for statistical computing and graphics. Besides standard R libraries, functions from package “compositions”, “robcompositions” were used [33–36]. Additionally function from package “cluster” were used in cluster analysis [37, 38].

## Model formulation and statistical methods

The soil horizon samples were collected in different places, which were not contaminated uniformly. It was obvious that location of the place from which sample was taken affects its total ^137^Cs activity. To avoid the effect of unequal initial soil contamination the relative activities *a*_r_ were calculated. The ^137^Cs activities *a*_*j*_ in consecutive soil horizons of the profile sample were added and then the activity *a*_*k*_ of each *k* horizon (*k* = 1..*j*) was divided by the sum of *m* horizons calculated previously.

1 $$ {a_{{{\text{r}}k}} = \frac{{a_{k} }}{{\sum_{j = 1}^{m} {a_{j} } }}} $$

The quantity *a*_r*k*_ describes the relative ^137^Cs activity in the *k*-th soil layer.

Formulation of the model of relationships between relative activity concentrations of ^137^Cs in soil was based on some assumptions. Primary influence of position of soil horizon in profile on *a*_r*k*_ was assumed. The soil horizons were represented by the numbered soil layers, in the sequence from surface towards deeper soil regions. In this way the differentiation of horizons occupying the same position in profile by different physicochemical properties vanishes, only information about layers sequences remains. Though this approach caused some information loss, it also simplified the structure of the considered soil profile. In this way the soil model was represented by the system of adjacent soil layers between which transport of ^137^Cs occurs.

The layers *w*_*j*_ were numbered starting from the soil surface down to the 5th soil horizon. In this system the layer can correspond to different soil horizons. The *w*_1_ and *w*_2_ layers corresponded to Ol and Of subhorizons respectively. These horizons were observed in all 39 soil profiles. Layer *w*_3_ was dominated by Oh (in 20 profiles) and A (15) horizons, though in 4 profiles it was AE horizon. Most of the *w*_4_ layers corresponded to A (17) and Bbr (15) horizons, but in some profiles Ees (5) or AE (2) also constituted this layer. The deepest layer, *w*_5_, mainly corresponded to Bbr (21) or C (16) horizon, only in 2 profiles it was the Ees horizon.

In the proposed description a certain direction of cesium transport between layers was not defined. The isotope could move from surface to deeper layers as well as in the opposite direction, i.e. from soil depth towards its surface. For such direction of soil solute transport the capillary forces can be responsible, in connection with water evaporation from the soil surface. Assumption concerning transport between successive, adjacent layers is not essential. For example, cesium from layer number 3 could be moved directly to layer 1, omitting layer 2. Among others, such phenomenon could be produced by plants. It should be observed when soil solution with dissolved ^137^Cs is transported from the soil layer where the roots are located to the parts of a plant which sprout above the ground. As a result of natural, seasonal life cycle ^137^Cs returns to soil with fallen leaves or fruits.

The proposed approach reflects a variety of possible cesium transport mechanism, which can include not only simple physiochemical transport in porous media but it can also be contributed by plants, microfauna, microflora and others.

Relationships between ^137^Cs concentration activities in soil layers constitute the foundation of the model. The proposed method of description of ^137^Cs distribution in soil is based on raw relationships between the experimental data rather than on the known physical, chemical or biological mechanisms. The certain ways of cesium transport, like diffusion, advection or bioturbation, are not explicitly included in model. Within the proposed model the results of analysis of relationships between ^137^Cs activity concentrations in soil layers deliver general information about transport directions between layers. This information might enable identification of an actual type of dominating transport mechanism and a route of cesium transport in soil as well as recognition of ^137^Cs state in chemical compounds comprising the layers. The prosed description enables comparison of the measurement results obtained for different soil types. It is also possible to estimate contamination with ^137^Cs of a region when only incomplete samples of soil layers are available.

The advantage of *a*_r_ utilization in description of ^137^Cs behavior in soil is impaired by specific properties of the studied variables. The compositional data calculated from Eq. 1 have specific properties. They are limited in the range from 0 to 1 and they are not independent on each other. Interpretation of such data needs special attention.

The constrained data which show compositions of a whole need proper statistical methods to avoid improper interpretation of the computation results and coming to false conclusions. The compositional data are not independent on each other, if content of one of components increases the others have to decrease. The particular properties of compositional data preclude the application of standard statistical techniques on such data in raw form. Usually the standard methods are designed for analysis of the data which are free to the range from −∞ to +∞ [39–43].

The results of compositional data analysis usually depend on selection of variables set taken for computations. Such effect is called subcompositional incoherence. This effect concerns, among others, the variability of the components. The variances and covariances of compositional vector components are not independent on each other. For this reason the results of standard statistical analysis of the relationships between raw components or parts are spoiled by spurious effects. There is no relationship between covariance structures, calculated for subcompositions which include common components. This incoherence disables, among others, proper inference about dependencies between composition components on the base of correlation coefficients.

In current analysis of data the methods resistant to subcompositional incoherence are used. It means that the type of the identified relationships between components remains unchanged even if a subset of components other than the analyzed ones, would be exchanged (the other subcomposition would be analyzed). Investigation of relationships between ^137^Cs relative activity concentrations in soil layers was based on the exploratory analysis of compositional data.

Sample space of compositional data variables is known formally as a simplex. In simplex space the structures are described by appropriate norm, scalar multiplication, distance between points, sum of two vectors and product of vector and number. Two operations defined in simplex space, perturbation and powering, are used in definition of linear process. The result of perturbation ⊕ of vectors ***x*** and ***v***, both of length *D*, is a vector of products of ***x***_*i*_ and ***v***_*i*_ (*i* = 1…*D*). Result of this operation corresponds to shift of point with coordinates ***x*** by vector ***v***. Powering ⊗ of vector ***v*** and a number *t* gives a vector, in which each component of ***v*** was raised to power *t*.

Both operations can be used in the linear process definition, using the parametric expression:

2 $$ {{\boldsymbol{x}}\left( t \right) = {\boldsymbol{x}}_{ 0} \oplus \left( {t \otimes {\boldsymbol{v}}} \right)} $$

where vector ***x***_0_ represents initial composition and vector ***v*** determines direction of composition changes in ***x***.

Compositional data contain only relative information. An approach based on log-ratios was developed, indicating that the relative magnitudes and variations of components, rather than their absolute values, should be used in computations of statistical parameters characterizing data. A number of methods were introduced which should be used in computations of statistical parameters characterizing data from the constrained sample space [43–46]. These methods include transformations of the constrained data to the unconstrained ones.

The compositional problems can be analyzed also in Euclidean space but the data have to be properly transformed. There are several transformation methods and each of them has its own drawbacks and advantages. In our computations the centered logratio (*clr*) transformation was used, which is defined for a vector ***x*** of non-zero compositions. In this transformation the logarithm of ***x*** component ratios to the geometric mean *g*(***x***) are calculated. Using *clr* transformed coordinates distance between two points can be calculated similar like in euclidean space. The sum of *clr* transformed variables is 0, which indicates that these coordinates are included in the same plane. The *clr* coordinate values depend on subcomposition, which has been taken for the calculation, i.e. the subcompositional consistency condition is not met.

Other method of compositional variable transfer from the simplex to the Euclidean space can be achieved using isometric logratio transformation. This transformation uses the orthonormal vector basis that allows direct mapping of distances and angles from the simplex into the Cartesian coordinate system [47, 48].

Another way to represent compositional variables in the system of orthonormal vector basis is construction of balances, which describe relationship between the components [49, 50]. This method involves division of the full composition on two disjoint subcompositions, which are used in balance creation. The components of these subcompositions can be selected using the procedure of sequential binary partition. This procedure is based on dividing, in successive steps, one subcomposition into two parts, which do not have a common component. One can mark components of these subcomposition, for example by “+” and by “−”. The first step is to provide two subcompositions. In next step the division is carried out using only one of them without the other. In this way, there are three subcomposition, two of them are used in balance creation and the third is ignored. If *m* is the number of components of a compositional variable, such division can be made *m*−1 times. In each step *l* a balance *z*_l_ can be computed from the relationship:

3 $$ z_{l} = \sqrt {\frac{rs}{r + s}} \ln \frac{{\left( {{{\Pi }}_{ + } } \right)^{ 1/r} }}{{\left( {{{\Pi }}_{ - } } \right)^{ 1/s} }}\quad {\text{for}}\;l = 1 ,\ldots ,D - 1 $$

where *r* is a number of components in subcomposition marked “+”, *s* is a number of components in subcomposition marked “−”, Π_+_ and Π_+_ are respective products of “+” and “−” components. The orthogonal base formed by balances is not unique. Other bases can be created using binary partition, for example, by permutations of components in composition.

Co-variability of two compositional vector variables ***x***_**A**_ and ***x***_**B**_ can be tested using variances of ratio *VR*:

4 $$ {VR = {\text{var}}\left( {{ \ln }\frac{{{{x}}_{{A}} }}{{{x}_{{B}} }}} \right)} $$

If a positive linear relationship between A and B compositions exists then ***x***_**A**_/***x***_**B**_ ratio is constant and its variance is 0. If there is a week or no relationship between ***x***_**A**_ and ***x***_**B**_ then the *VR* value grows up.

For the data structure analysis the cluster analysis methods were used. These methods allow to assign objects to different groups, so that the data in each subset share some common trait. For each pair of compositional points the distance between them in simplex space was calculated. The distances were then used in cluster analysis.

An overview of cluster existence in data is shown on dendrograms—a tree diagrams illustrating the arrangement of the clusters. Diagrams were constructed using “agnes” and “diana” functions from “cluster” library. The first of the functions uses agglomerative nesting procedure and the second one applies divisive method [36, 37].

For multivariate data investigation the principal component analysis (PCA) procedure was utilized. PCA uses orthogonal transformation to convert a set of observations into a set of values of linearly uncorrelated variables. This method involves calculation of the eigenvalues or singular value decomposition of covariance or correlation matrix computed from data. Because of the relations between variances and covariances in compositional data the PCA results obtained for raw compositional data would be confusing. To avoid this effect in PCA the *clr* transformed data were used.

Occurrence of outliers in the data, i.e. the points strongly influencing the results of calculations, was expected. Utilization of robust statistical methods allow to reduce impact of these observations on the results of calculations, enabling formulation of appropriate conclusions concerning data structure and relationships between variables [51, 52]. For identification of outliers the estimator of the minimum covariance determinant (MCD) was calculated, which value was computed from the *ilr* transformed data [53, 54]. In MCD method a subset of all the data is selected, for which the determinant of the covariance matrix estimator is as small as possible.

## Results and discussion

The ^137^Cs activity concentrations were related to the localization of sampling site and on the depth from which soil sample was taken. Usually the biggest values were observed at the bottom of organic horizon, close to the region where mineral horizons start to appear. The smallest measured value of ^137^Cs specific activity concentration was 0.01 Bq/kg, and the biggest one was nearly 4 kBq/kg. The interquartile range was limited between the values of 18.3 and 1.04 kBq/kg. The estimators of the data center of specific activity concentrations were 636 Bq/kg (arithmetic mean), 92.6 Bq/kg (geometric mean) and 119 Bq/kg (median).

In calculations of the relative ^137^Cs activity concentrations Eq. 1 was used. The basic statistical parameters of *a*_r_ values in soil layers are shown in Table 1.

**Table 1:** The statistical parameters of the *a*_r_ values calculated for *w*_1_..w_5_ soil layers

	*w*_1_	*w*_2_	*w*_3_	*w*_4_	*w*_5_
*min*	0.003	0.113	0.020	0.000	0.000
*q*_1_	0.026	0.275	0.302	0.011	0.002
*Me*	0.035	0.365	0.449	0.051	0.003
*a*_r_	0.046	0.420	0.421	0.103	0.010
*g*_*a*r_	0.034	0.379	0.365	0.031	0.003
*q*_3_	0.063	0.523	0.516	0.161	0.009
*max*	0.258	0.971	0.769	0.404	0.120

In the Table the *min* and *max* parameters are minimum and maximum of *a*_r_ values respectively, *q*_1_ and *q*_3_ are lower and upper quartile, *a*_r_ is arithmetic mean, *g*_ar_ is geometric mean and *Me* is median

The data presented in Table 1 show that the biggest relative ^137^Cs activity concentrations were observed in w_2_ and w_3_ layers. Also range of *a*_r_ changes in these layers was relatively big, comparing to the other layers.

**Fig. 1:**
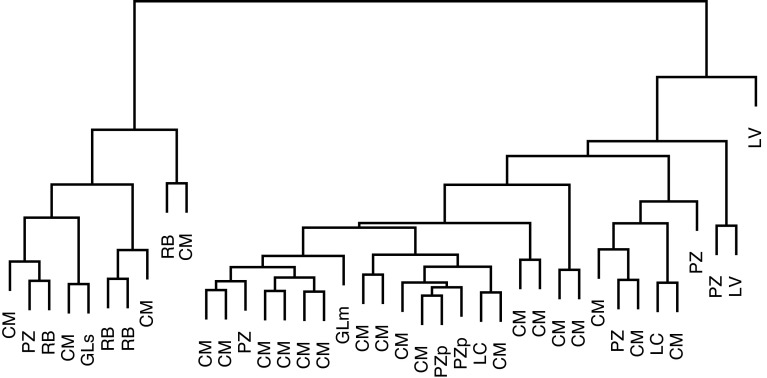
Structure of the dendrogram describing relative activities in soil layer constructed using divisive algorithm

An assessment of the data structure was performed. In space of relative activity concentrations, location of each point representing 1 of 39 soil profiles was described by the *w*_1_..*w*_5_ variables. In first step of analysis the matrix of distances in 5D simplex space between all pairs of points was calculated. The matrix was used in construction of dendrograms describing ^137^Cs relative activities in soil profiles. Both divisive and agglomerative clustering methods were used. Additionally each branch of dendrogram was marked by symbol of the soil type from which the sample of profile was taken. The structures of dendrograms were similar and as an example the one constructed using divisive algorithm is shown in Fig. 1.

Independently on the clustering method used, in dendrograms two main clusters can be observed. The smaller one of them occupies approximately ¼ of the area in left side of dendrograms. In both clusters similar frequencies of most of soil types representation could be observed. Though the RB soil type appears only in one cluster, the low number of such profiles precludes valid inference concerning incidental occurrence of this soil type in a single group. The observation suppose that the distribution of relative ^137^Cs concentration activities in soil profile does not clearly depend on soil type. Finally, no predominance of the certain soil type (or types) representation in two branches could be assumed. Though the reason why the groups appear currently remains unknown, the dendrogram structure supposes no influence of soil type on clustering. This conclusion supports disregarding of soil type in data analysis.

**Fig. 2:**
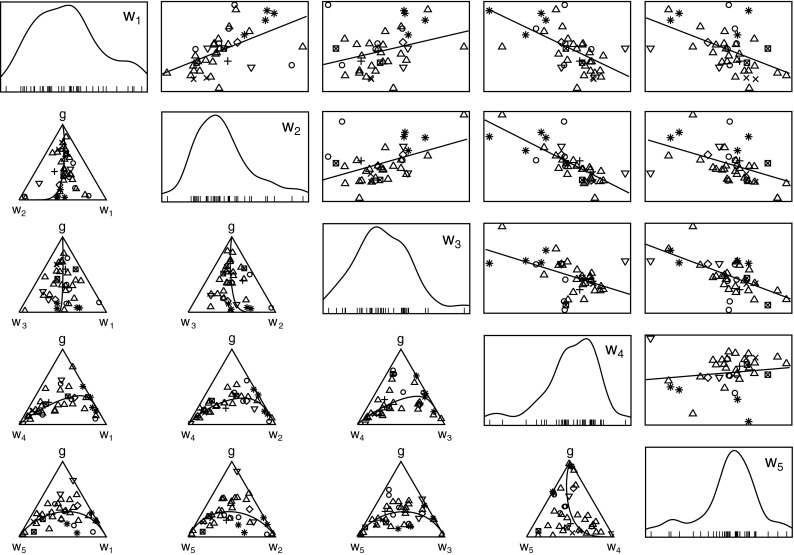
Relationships between relative ^137^Cs concentrations of activity in soil layers *w* shown in simplex space (raw data) and in cartesian coordinates (*clr* transformed data). In the diagonal the density plots of transformed data are plotted. The points corresponding to different soil types are marked with different symbols: CM *triangle*, LC *squared times*, PZ *open circle*, GLs *diamond*, RB *eight pointed black star*, *LV* *inverted triangle*, GLm *plus* and PZp *times*

In Fig. 2 the relationships between relative ^137^Cs concentrations of activity in soil layers *w*_*j*_ (*j* = 1…5) are shown. For comparison the relationships between raw data in simplex space (below diagonal) and *clr* transformed data in Cartesian coordinates (above diagonal) are shown. Solid lines describe changes in compositions modeled by linear process in simplex space and linear relationship between transformed variables in Euclidean space, respectively. In the diagonal the graphs of density plots of transformed data are also shown.

The analysis of relationships between the relative activity concentrations of ^137^Cs in different soil layers must take into account both the graphs showing the raw data in the simplex and the transformed data shown in Euclidean space. The off-diagonal graphs show more or less clearly outlined relationships between the variables studied. The co-variabilities of ^137^Cs relative activity concentrations in soil layers, estimated by *VR* coefficients, are shown in Table 2.

**Table 2:** Variances of logratios *VR* calculated for pairs of relative ^137^Cs concentrations in soil layers *w*

	*w*_1_	*w*_2_	*w*_3_	*w*_4_
*w*_2_	0.85			
*w*_3_	1.2	1.1		
*w*_4_	5.1	6.2	5.2	
*w*_5_	3.8	4.1	4.4	3.8

The linear relationship between *a*_r_ in *w*_1_ and *w*_2_ is well-defined. Co-variability of these variables shown in the simplex and, after the transformation, in Cartesian coordinates suggest proportionality of their values. The calculated geometric mean of the ratio *a*_r,1_/*a*_r,2_ was 0.090. Proportionality confirms the smallest value of *VR* parameter in Table 2. Reasons of a constant proportion of ^137^Cs content in the layers *w*_1_ and *w*_2_ may be different. This might be a result of much stronger retention of ^137^Cs in *w*_2_ than in *w*_1_, linked to the existence of different chemical compounds in these layers. It is also possible that the amount of ^137^Cs in *w*_1_ and *w*_2_ is similar, but due to reduced weight of the other components of the system concentration of the ^137^Cs increases. The *w*_2_ layer consists mainly of organic matter which is much more decayed than in *w*_1_. Such decomposition is accompanied by emission of chemical compounds that are volatile or are weakly bound to solid components of the layer. The result of such decomposition is loss of mass of the entire system causing increase in ^137^Cs content which is bound with the components immobilized in the layer.

Comparison of the relative activities of ^137^Cs in layers *w*_2_ and *w*_3_ shown that they are very similar. Their geometric mean of ratios is 1.0. This suggests similar properties of the two layers, which affect the ^137^Cs accumulation or similar mechanisms of their formation process. The average value of *a*_r,1_/*a*_r,3_ ratio was 0.094, which confirms the approximately nine times lower content of ^137^Cs in the surface layer compared to the ones located somewhat deeper.

The results of the calculation can also be interpreted using a linear model in the simplex, formulated by Eq. 2. It can be assumed that the observed changes in composition of the system are the result of the occurrence of a process. The stage of the process progress is different in the studied soil samples, although the mechanism is fixed. This mechanism is determined by the direction of the composition changes in the individual layers defined by vector ***v***. The values of vector ***v*** components calculated for *w*_1_ and *w*_2_ are ***v***_1,2_ = [0.39, 0.48, 0.13]. In this vector, the first two components determine the change in the relative activity concentrations of ^137^Cs in the first and second layer, a third component describes the change in the geometric mean of relative activity concentrations calculated for the remaining layers. The components of the vector describing the direction of ^137^Cs content changes in *w*_2_ and *w*_3_ are defined by ***v***_2,3_ = [0.45, 0.42, 0.13]. Similar to ***v***_1,2_ are the components of vector ***v***_1,3_ = [0.38, 0.49, 0.13]. Although the layer *w*_1_ and *w*_3_ are not in direct contact with each other, the mechanism of ^137^Cs transport between them appears similar to the same mechanism for *w*_1_ and *w*_2_. Uniform contents of ^137^Cs in *w*_2_ and *w*_3_, and nearly the same components of vectors ***v***_1,2_ and ***v***_1,3_ suggest considerable similarity of the two layers, affecting the transport of ^137^Cs between them and *w*_1_.

Graphs shown in Fig. 2 suggest a linear nature of the transfer process of ^137^Cs between *w*_4_ and the layers above. In particular, it is clearly visible for the layers *w*_2_ and *w*_4_. The following values of vector ***v*** components were calculated: ***v***_1,4_ = [0,48; 0,13; 0,40], ***v***_2,4_ = [0,51; 0,13; 0,36] and ***v***_3,4_ = [0,48; 0,13; 0,39]. It can be noticed that the ***v*** vectors are similar. As for layers 1–3 one can assume that the same mechanism controls the transport of ^137^Cs between the fourth layer and the layers located above.

In comparison to *w*_4_, relationships between relative activity concentration of ^137^Cs in *w*_5_ and activities in other layers is outlined less clearly. However, as for *w*_4_, relationship between the relative activity of ^137^Cs in *w*_5_ and the activities in *w*_1_, *w*_2_ and *w*_3_ are discernible. The following values of components of vector ***v*** were calculated: ***v***_1,5_ = [0,49; 0,13; 0,38], ***v***_2,5_ = [0,52; 0,13; 0,35] and ***v***_3,5_ = [0,52; 0,13; 0,35]. The components of ***v*** are very similar to their counterparts calculated for *w*_4_ layer. It can be assumed that the same mechanism is responsible for ^137^Cs transport between the surface and *w*_4_ as well as *w*_5_ layers.

There is no significant association between *a*_r_ in layers *w*_4_ and *w*_5_. These are the only among adjacent layers in which the relative activity concentrations of ^137^Cs were unrelated.

**Fig. 3:**
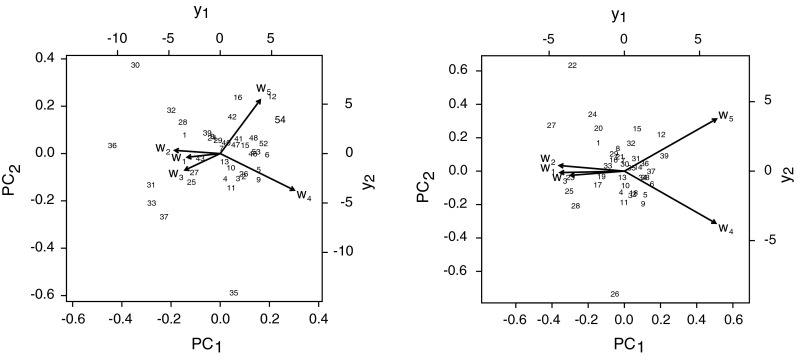
Biplots of structure of the first two principal components PC_1_ and PC_2_ and the results of data projection onto the plane formed by these components. Graph on the *left* shows the results obtained by the standard PCA, and graph on the *right side* shows the results obtained by robust method

For analysis of reciprocal relationships between relative ^137^Cs activity concentrations in soil layers the PCA method was used. Due to the scattered points clearly visible in the graphs it could be expected occurrence of outlying observations in the data. For this reason both traditional and robust methods were used in data analysis. Figure 3 shows the structure of the main components and the results obtained with the standard PCA method (left column) and with the robust PCA method (right column).

The first two principal components, calculated using the standard method, contain 87 % of variability of the whole, as calculated by robust method they contain 91 % of the variance. It is easy to note that the two graphs shown in Fig. 3 are very similar. Application of the robust PCA practically does not change structure of the graph, comparing to the standard PCA. Though the interpretation of biplots constructed from transformed compositional data is somewhat different from the ones for unconstrained data in Euclidean space [55], some conclusions can be drawn. There is a clear relationship between the components representing relative ^137^Cs concentration of activities in *w*_1_, *w*_2_ and *w*_3_, suggesting increase in the value of one variable with increase in value of the other. This observation is consistent with the conclusions previously reached on the basis of *VR* values, shown in Table 1.

Among the variables representing the relative content of ^137^Cs in surface soil layers and layers lying deeper there is no simple relationship. Large mutual distance between the arrowheads representing a group of components *w*_1_-*w*_3_ and *w*_4_ and *w*_5_ shows the lack of a positive covariability between *a*_r_ in these layers.

In further analysis of the measurement results, the balances defined in Eq. 3 were used. There is a number of different choices of a particular set of balancing variables in description of the system. They can be chosen in a certain manner to describe previously identified processes or can be used in exploratory analysis of compositional variables. To the balance a physicochemical interpretation could be assigned, which is derived from its mathematical form. The main part of Eq. 3 is constituted by the ratio of concentrations products. If a reversible chemical process occurs then concentrations of the substances involved in such reaction are related with each other. Ratio of concentration products in numerator and denominator is constant at the given physical conditions. In opposite, the variability of concentration ratios of the processes far from equilibrium state would be big since the ratios values are different at various reaction progress.

In the absence of initial assumptions concerning the relationship between the relative activity concentrations of ^137^Cs in each layer, all possible balances consisting of 2–5 components were taken into account. A total number of 90 different balances were constructed. The coordinates of points representing results of the measurements were projected onto the base vector representing a single balance, and then the variance of the points positions, localized in the new coordinate, was calculated.

Variances of balances were limited in the range from 0.43 to 4.2. They depend mainly on the components making up the balance, but one can also notice a tendency to increase in variance with the number of components in balance.

**Fig. 4:**
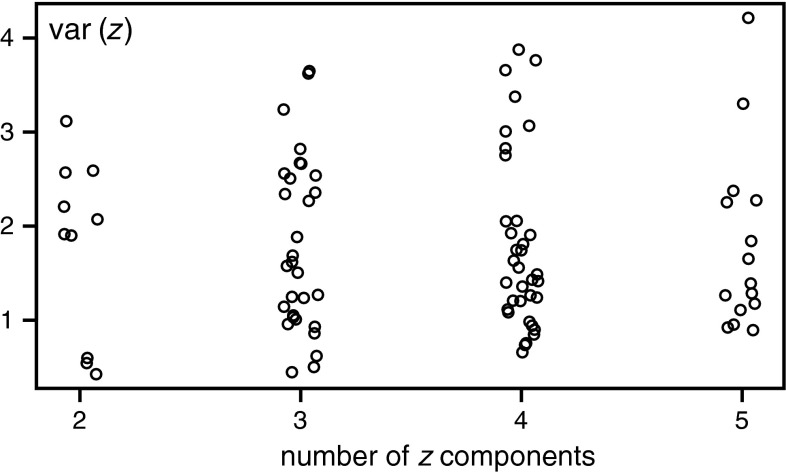
Variances of balances calculated for subcompositions with different numbers of components

In Fig. 4 the calculated variances of balances, composed of different numbers of components, are shown. In order to avoid overlap between the points in the graph, to the abscissa of each point a small random value was added.

In Table 3 the parameters describing balances *z* and their variances are presented. Only the balances with the small and big variances are shown. The *l*_s_ parameter is the number of components used to create a balance, *g*_+_ and *g*_−_ determine the products of the components, respectively in numerator and denominator of the balance. For clearer presentation, the cursive style in layers symbol and layers numbering by subscript were abandoned. Symbols of layers in the groups *g* are separated by “·”.

**Table 3:** The parameters describing balances *z* and their variances

var(*z*)	*l*_s_	*g*_+_	*g*_−_
0.43	2	*w*_1_	*w*_2_
0.45	3	*w*_2_	*w*_1_·*w*_3_
0.50	3	*w*_2_·*w*_3_	*w*_1_
0.54	2	*w*_2_	*w*_3_
0.60	2	*w*_1_	*w*_3_
0.62	3	*w*_3_	*w*_1_·*w*_2_
0.66	4	*w*_1_	*w*_2_·*w*_3_·*w*_5_
…	…	…	…
3.38	4	*w*_1_·*w*_3_	*w*_4_·*w*_5_
3.62	3	*w*4	*w*_2_·*w*_3_
3.65	3	*w*4	*w*_1_·*w*_2_
3.66	4	*w*_1_·*w*_2_	*w*_4_·*w*_5_
3.76	4	*w*_2_·*w*_3_	*w*_4_·*w*_5_
3.88	4	*w*_4_	*w*_1_·*w*_2_·*w*_3_
4.21	5	*w*_4_·*w*_5_	*w*_1_·*w*_2_·*w*_3_

The *l*_s_ parameter is the number of components used to create a balance, *g*_+_ and *g*_−_ determine the products of the components, respectively in numerator and denominator of the balance

It can be seen that the balances with the smallest variances are constructed from the relative activity of ^137^Cs in layers located close to the surface, i.e. *w*_1_, *w*_2_ and *w*_3_. Relatively small variations of those balances suggests low variability of two products ratio. This could be a result of thermodynamic equilibrium of processes responsible for the ^137^Cs transport in the system of three layers, described by the law of mass action. Selection of the certain balance with low variance, as a hint to help determine the mechanism of the process, is ambiguous. The almost constant value of the concentration products ratio can be noticed in process that are described by *w*_1_ *w*_3_/*w*_2_ and *w*_2_ *w*_3_/*w*_1_. Also variability of *w*_1_ *w*_2_/*w*_3_ is only slightly higher than the previously described. The relatively low variability ratios also show some of the products comprising four components. However, here also it is difficult to identify such unique balance structure for which variability is the lowest.

The biggest variance was calculated for balance composed of five components which are divided into two groups, surface components (*w*_1_, *w*_2_, and *w*_3_) and the ones located deeper in soil profile (*w*_4_ and *w*_5_). Somewhat smaller variances have balances composed of three or four components. The common feature of these balances is their characteristic structure. Similarly like in the balance with the highest variance, here also are two groups of components, the surface ones and the ones located deeper.

The described method of data elaboration and modeling of ^137^Cs distribution in soil demonstrates its particular advantages. In this approach strict assumptions, like transport direction or its physical mechanism, are not required. The method can be used in extraction of unique information from measurements results. The assessment of components of directional vector ***v*** allows to estimate character of processes affecting ^137^Cs distribution in soil layers. From the results of the balances ***z*** analysis, the thermodynamic characteristics of processes can be deduced. The method of data elaboration, applied currently in analysis, allowed to discover character of processes controlling ^137^Cs behavior in soil profiles. It was supposed that sequence of soil layers, irrespective or nearly irrespective to their detailed physicochemical characteristics, determines ^137^Cs distribution in soil profile. Though detailed mechanisms of processes remain unknown, the results obtained could be used in further studies.

## Conclusions

In this paper mutual relationships between relative ^137^Cs activity concentrations in soil layers were described and discussed. The structure of soil profile described by separate genetic horizons, appropriate for the soil subtype, was considerably simplified. In analysis soil horizons were represented by layers. These layers were numbered in the order in which they form a soil profile, hence the informations regarding soil subtype and genetic soil horizon type have been dropped. Though the physical and chemical properties of genetic soil horizons have a significant impact on accumulation of ^137^Cs, the applied simplification did not lead to loss of information, which is necessary to describe the phenomenon. Results of the calculations confirmed the effectiveness of such an approximation. The relationships found in data analysis are related to the phenomenons which are not evidently linked to physicochemical properties of soil horizon.

The analyzed data were specific. They were represented by compositional variables whose components are the relative contents of ^137^Cs in the soil layers. Since the sum of the components in each soil profile was 1, the sample space was limited in the appropriate simplex. For this reason in data processing the proper methods, designed for this type of variables analysis, were used.

The results of analysis showed that the relationships between the relative activity concentrations of ^137^Cs in soil layers, due to their nature, might be divided into two groups. The first of them was related to layers *w*_1_, *w*_2_ and *w*_3_. The *a*_r_ values in these layers are proportional to each other, and ^137^Cs distribution mechanism within them has the characteristics of the process leading to thermodynamic equilibrium. The second group concerns layers that are located deeper, i.e. *w*_4_ and *w*_5_. The calculation results suggest lack of thermodynamic equilibrium between these layers and layers located above. Using a linear model to describe the *a*_r_ changes in *w*_4_ and *w*_5_ one could conclude that these alterations occur much faster in layers lying above *w*_4_.
